# Vibrational Fingerprinting of Gas Mixtures Using COCO-QEPAS

**DOI:** 10.3390/s26030846

**Published:** 2026-01-28

**Authors:** Simon Angstenberger, Emilio Corcione, Tobias Steinle, Cristina Tarin, Harald Giessen

**Affiliations:** 1 4th Physics Institute and Research Center SCoPE, University of Stuttgart, Pfaffenwaldring 57, 70569 Stuttgart, Germany; simon.angstenberger@pi4.uni-stuttgart.de (S.A.); t.steinle@pi4.uni-stuttgart.de (T.S.); 2 Institute for System Dynamics, University of Stuttgart, Waldenburgstrasse 17, 70563 Stuttgart, Germany; e.corcione@isys.uni-stuttgart.de (E.C.); cristina.tarin-sauer@isys.uni-stuttgart.de (C.T.)

**Keywords:** QEPAS, photoacoustics, trace gases, OPOs, linear regression, gas mixtures, coherent control

## Abstract

Detection and simultaneous monitoring of multiple trace gases is vital in scientific and industrial processes. Here, we use coherent control in quartz-enhanced photoacoustic spectroscopy (COCO-QEPAS) with an in situ learning method for rapid fingerprinting of trace gases to identify and monitor arbitrary gases at very low concentrations, without prior knowledge of gas composition. We validate this on various mixtures, including CH_4_/C_2_H_2_/C_2_H_4_/C_2_H_6_/NO_2_/NH_3_. To this end, we demonstrate real-time analysis of mixtures containing up to four trace gases at ppm-level, monitoring changes in seconds using linear regression. The scalability of simultaneously distinguishable gases is straightforward. Furthermore, we expand fingerprinting to 10 ppm with a detection limit of 180 ppb CH_4_, and apply empirical mode decomposition as an adaptive, data-driven filtering method to recover characteristic spectral features at the noise floor. For quantitative analysis in the ppb regime, we employ principal component regression as a calibration model that exploits correlations across the full spectrum. Consequently, our method offers significant potential for sensing applications where speed, accuracy, and simplicity are critical.

## 1. Introduction

Gases form the human environment and are utilized in a wide range of applications in both research and industry. They shape the Earth’s environmental conditions [1], are used as catalysts in industrial processes [2,3,4] and reveal chemical reaction dynamics [5,6]. Whilst gases are used in numerous ways, many of them are toxic or harmful to the environment. At the same time, they are almost impossible to detect by relying purely on human senses.

Gas detection is therefore realized in many ways: mass spectrometry samples by the atom weight [7], gas chromatography samples by phase separation [8], and micro-cantilever microelectromechanical systems and plasmonic nanoparticles by adsorption [9,10,11,12]. Beyond that, most molecules substantially absorb light in the mid-infrared through rotational-vibrational transitions. These transitions can be excited when shining light onto the sample that matches the respective frequencies. As the rotational and vibrational frequencies directly depend on the atomic masses and species involved, the shape of the absorption over a certain wavenumber range is specific and unique for each molecule. Therefore, the absorptive shape is called “fingerprint” [13] as it allows to directly deduce the kind of molecule present in the sample using the HITRAN database [14,15].

Various techniques can detect this absorption: direct absorption spectroscopy (DAS) and interferometric Fourier-transform infrared spectroscopy (FTIR) are straightforward [16,17,18]. These two methods show the fundamental conflict of gas spectroscopy: The former has good power per line while being limited in its detection range. The latter has a high detection range, but at the cost of optical power per absorption line. To enhance the detected signal, cavity ringdown spectroscopy (CRDS) [19] uses an increased absorption path length. Advanced approaches combine multiple light sources such as dual-comb spectroscopy (DCS), which allows for low detection limits and broadband detection of multiple species simultaneously [20,21,22,23]. However, these advantages come in at high economic cost and large detection volumes. Hence, photoacoustic spectroscopy (PAS) has gained widespread use, offering low-cost detection and small sampling volume [24,25,26]. In its more sophisticated variant, quartz-enhanced photoacoustic spectroscopy (QEPAS), the detection volume is reduced even further [27,28,29].

Although the photoacoustic effect has been known for decades [30], it is the advent of high-power, narrowband, and stable laser sources that has rendered it a powerful tool in gas diagnostics [31,32]. The signal-to-noise ratio (SNR) is usually high, and detection limits reach the parts-per-billion (ppb) [33,34,35] or even parts-per-trillion range [36]. This is possible because a wavelength- or intensity-modulated beam is resonantly enhanced in the detector.

However, typically, only a single absorption line is resolved. Diode lasers are the most common laser source, especially in wavelength-modulation techniques, and their tuning range is limited to a few nanometers. When multiple gases are present in the sensing volume, substantial effort is required to distinguish between them [37,38]. Often, external cavity diode lasers (ECDL) benefitting from external gratings as tuning elements [39] and quantum cascade laser (QCL) batteries [40,41,42] are used. Widely tunable laser sources are a possible tool to overcome this limitation as it is possible to resolve many absorption lines or even the entire fingerprint region [43,44,45,46,47].

Broadband conventional photoacoustics was already demonstrated [47,48]. However, the detection volume in the resonator is usually large and external noise contributions are picked up. The smaller sensing volume of QEPAS cells (typically single-digit cm^3^) allows faster response times to changes in the gas composition with a rapid transient response [49]. Also, only a relative movement of the prongs to each other gives a detectable voltage, making it robust against external noise contributions compared to conventional photoacoustics [50]. Most studies of gas detection using QEPAS are investigating a single gas, because only a single wavelength is available. Simultaneous monitoring of multiple gases within a short timeframe has barely been investigated. Some values of recent QEPAS results are listed in Table 1 with a focus on detection limit and the sampling time. Detection limits usually reach the ppm to ppb regime and detection times are often only a few milliseconds up to hundreds of seconds when finding the ultimate limit of detection is the primary goal. For this work, we state a range of values and will discuss the optimal parameter range later on in the manuscript.

Here, we demonstrate simultaneous concentration monitoring of multiple trace gases on a timescale of seconds. Furthermore, we apply a transient photoacoustic technique, namely COCO-QEPAS, to sample fingerprints of methane in the ppb-region and demonstrate trace gas measurements of CH_4_, C_2_H_2_, C_2_H_4_, C_2_H_6_, NO_2_, and NH_3_. This shows that the setup can sense arbitrary trace gases if they are infrared-active due to the wide wavelength range and quick tunability of the laser source. We demonstrate that linear regression is a simple tool to decompose photoacoustic signals into individual concentration contributions. In the future, latent structures regression could make the sensing capabilities more robust as it has been proven as a reliable gas detection regression candidate [51].

Our results indicate that COCO-QEPAS is a powerful tool to quickly analyze complex gas mixtures in a dynamic range from ppb to % at low cost and low sampling volumes. This will further boost the progress of QEPAS as a spectroscopic sensing technique in all areas of science and technology that employ gases at concentrations fluctuating strongly over time.

## 2. Materials and Methods

**Figure 1:**
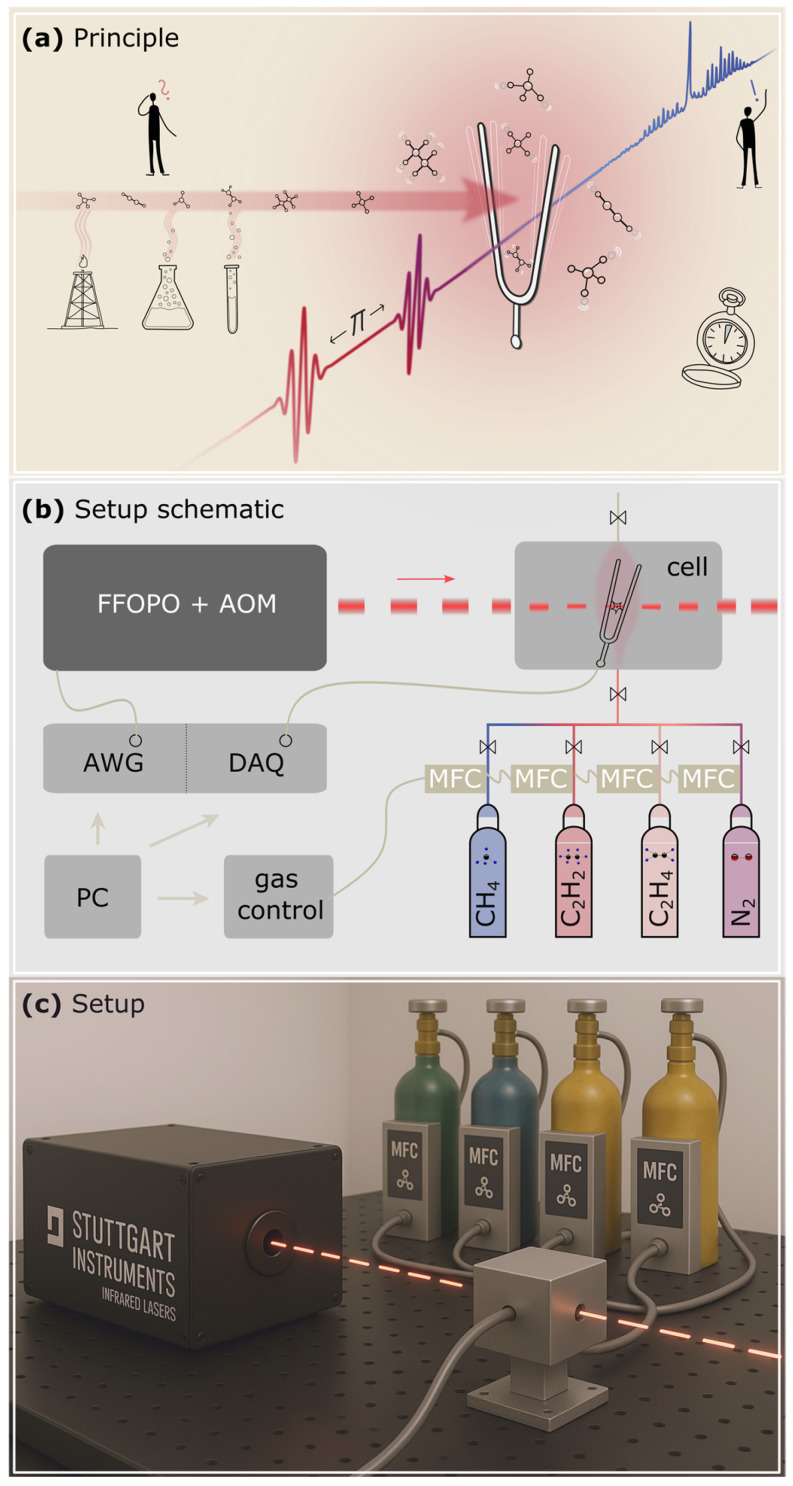
Real-time trace gas analysis of arbitrary gas mixtures using COCO-QEPAS. (**a**) On the left, sources of complex gas mixtures are highlighted. Decomposition of the absorption spectrum (blue) enables independent concentration retrieval over multiple orders of magnitude (ppb to %). (**b**) Schematic of the setup in a laboratory. A widely tunable fiber-feedback optical parametric oscillator (FFOPO) feeds a QEPAS cell. An acousto-optic modulator (AOM), controlled by an arbitrary waveform generator (AWG), tweaks the laser bursts. Data acquisition (DAQ) is performed on the same device. Four different gases can be fed into the cell, controlled by mass flow controllers (MFCs). (**c**) Simplified real-life laboratory setup.

The experimental principle, setup, and some potential applications are illustrated in Figure 1. In panel (a) of Figure 1, various trace gas sources are sketched on the left, with an emphasis on hydrocarbons. They have a high abundance in almost all chemical reactions ranging from petrochemical processes and analytical chemistry applications to reaction control. Conventional non-destructive, real-time analysis of mixtures usually requires large-scale and expensive instrumentation.

The primary detection element is a quartz tuning fork (QTF). When a molecule is absorbing light, the absorbed energy is re-emitted as heat which creates a pressure wave acting on the prongs of the fork. Subsequent laser pulses (red) can resonantly enhance the detection. A second burst of pulses, shifted by Pi with respect to the QTF oscillation, can then decelerate the QTF. This enables a fast measurement at the next spectral data point. In this manner, spectral fingerprints (blue) are obtained within three to twelve seconds (depending on the swept wavelength range) through continuous sweeping of the laser wavelength Individual signal readout at each wavelength takes 20 ms. Details of this method, including number of excitation cycles and the optimal timing of the phase shift have been described in [46]. The calculation of the normalized noise equivalent absorption (NNEA) of the system to 3.7 × 10^−9^ W cm^−1^ Hz^−1/2^ can also be found in [46].

The schematic of the laboratory setup is laid out in Figure 1b. Sequence control via an arbitrary waveform generator (AWG) and data acquisition (DAQ) using lock-in detection are integrated into a single device (UHFLI, Zurich Instruments, Zurich, Switzerland). In this manner, both AWG and DAQ are synchronized to the same internal oscillator clock operating at 12,420.4 Hz. The integration time was set to 400 µs at a filter order of 3, corresponding to 5 oscillations of the fork. A laptop (PC) controls the UHFLI via an application programming interface. It also steers the gas flow in the mass flow controllers (MFC) for gas mixing. Two of the MFCs have a maximum flow rate of 2 L min^−1^ and the other two have a maximum flow rate of 250 mL min^−1^. This enhances the accessible mixing ratios of the gases. The gases are subsequently sent into the cell, controlled by individual valves. The laser pulses are generated by means of an acousto-optic modulator (AOM) from a fiber-feedback optical parametric oscillator (FFOPO; Piano, Stuttgart Instruments, Stuttgart, Germany) with a linewidth of 2 cm^−1^ and an average optical power of 100 mW.

To complement the schematic drawing of the setup, a 3D-rendering is displayed in Figure 1c. A laser burst (red) from the FFOPO (black) hits the QEPAS detection cell (Thorlabs, ADM01, Munich, Germany) which is fed by gas bottles and MFCs. For the sake of clarity, two beam-steering mirrors and a focusing CaF_2_ lens (f = 75 mm) are omitted from the drawing. The MFCs and gas bottles can be exchanged with ease, allowing for measurements of different gases.

## 3. Results and Discussion

**Figure 2:**
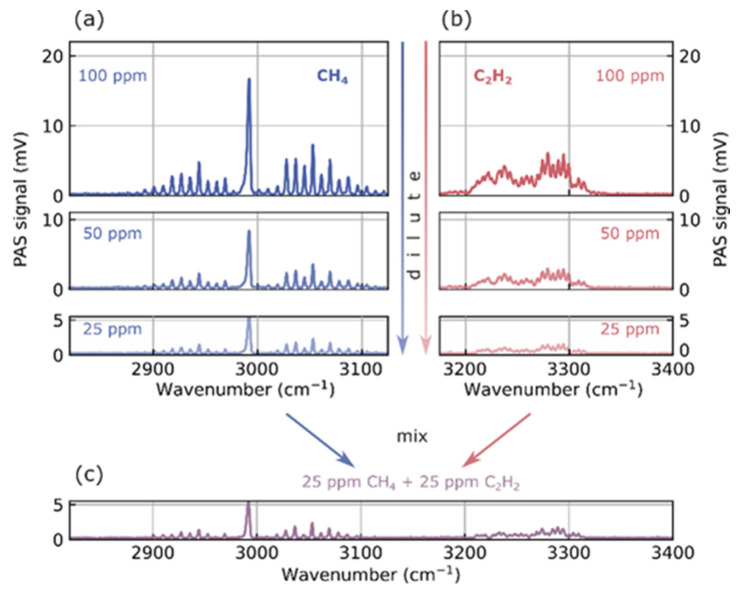
Measurement of diluted gases and their mixtures. Panel (**a**) displays the measurement of diluted methane (CH_4_) at decreasing concentration. In turn, the magnitude of the measured PAS amplitude decreases. In (**b**), the same procedure is performed for Acetylene (C_2_H_2_) with the same decreasing behavior. As the measurement setup allows mixing of different trace gases, we depict their combined measurement at a concentration of 25 ppm each in (**c**).

Ppm-level spectra of trace gases can be acquired by COCO-QEPAS in a large wavenumber range with this setup, owing to the gap-free large tunability of the laser source from 1.4 µm to 5 µm. It is also possible to mix several gases and to distinguish them as displayed by Figure 2.

The panels in Figure 2a depict acquired spectra for methane in blue with decreasing concentration from top to bottom. The magnitude of the photoacoustic signal decreases linearly with decreasing concentration. This is visually indicated by less opaque lines of the corresponding spectra for 100 ppm, 50 ppm, and 25 ppm, respectively. The tuning range of the laser source is not limited to the methane fingerprint region. Thus, a second gas, acetylene (C_2_H_2_) is shown in red in Figure 2b on the extended tuning range. Acetylene has only a small spectral overlap with the methane fingerprint. In a similar fashion to (a), the magnitude of the spectra decreases with decreasing amplitude from top to bottom. Decreasing the trace gas concentration is possible by connecting one MFC to pure N_2_ and adjusting the ratio of trace gas and N_2_ flow rates.

In this case, it is easy to sense both gases simultaneously by extending the measurement range. The plot in Figure 2c shows the simultaneous measurement of two gases at the same time for a concentration of 25 ppm each of methane and acetylene. Features of both gases are resolved with the same magnitude and linewidth as the individual measurements in the plots directly above the mixture. The spectra are averaged over three sweeps, as this plot is solely intended to illustrate gas dilution and mixing. This prevents the reader from being distracted by noise features in the data. Therefore, the total acquisition time of each averaged spectrum is approximately 20 s.

However, the large tuning range of the laser allows sensing of even more gases, even when they have spectral overlap. One tool to take all available data into account is linear regression. For a single gas, the photoacoustic spectrum at a given concentration *c* can be described by a one-dimensional vector $\overset{⃑}{S(c)}$. Each entry in the vector corresponds to an absorption value at one wavenumber point. In other words, each spectrum with *n* data points is a vector with *n* entries. Now, we can relate this spectrum $\overset{⃑}{S(c)}$ at a concentration *c* to a reference measurement vector $\overset{⃑}{S_{\mathrm{ref}}}$ at a known concentration $c_{\mathrm{ref}}$ via

(1) $$\overset{⃑}{S(c)}=\frac{c}{c_{ref}}\;\cdot \;\overset{⃑}{S_{ref}}.$$

Here, we assume that the signal strength, i.e., the absorption, scales linearly with concentration. The vector contains *n* data points, so this is an over-defined linear equation system and therefore solvable. As we have *n* data points available, we can easily include multiple gases in the linear regression solver. Equation (1) for *m* gas species denoted with index *i* then reads

(2) $$\overset{⃑}{{S(c}_{i})}=\sum_{i=1}^{m}\frac{c_{i}}{c_{i,ref}}\;\cdot \;\overset{⃑}{S_{i,ref}}.$$

As long as the number of gases *m* is smaller than the number of measurement points *n*, i.e., *m* < *n*, linear regression can minimize the square sum of deviation and retrieve concentration values.

**Figure 3:**
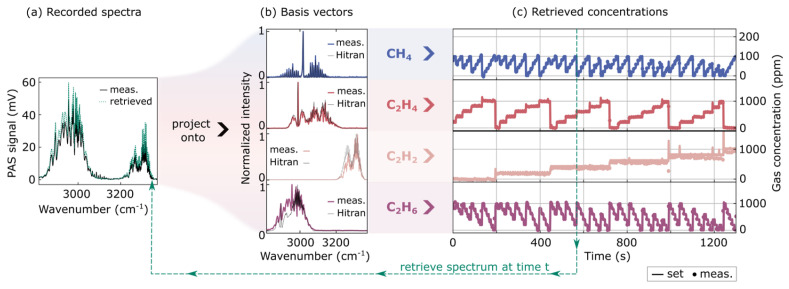
Real-time concentration analysis of a mixture of petrochemical trace gases. The mixture contains CH_4_, C_2_H_2_, C_2_H_4_, C_2_H_6_, diluted in a N_2_-matrix at various concentrations. Panel (**a**) displays measured (black) and retrieved (green) absorption spectra. The measured spectra are then mapped to reference spectra of trace gases at known concentrations, depicted in (**b**). Theoretical spectra from the HITRAN database are indicated in black for comparison. The result of this linear regression is shown in (**c**) as concentration of the gas. We show over 100 possible combinations here within a measurement time of over 1200 s (>20 min). A retrieved spectrum from the concentrations is plotted in green as visual comparison.

Consequently, parallel measurements on four gases are depicted in Figure 3. Here, the gases for testing are methane (CH_4_) at 200 ppm and acetylene (C_2_H_2_), ethylene (C_2_H_4_), and ethane (C_2_H_6_) each at 2000 ppm. During the experiment, methane is diluted between 0 ppm and 100 ppm, and the other gases from 0 to 1000 ppm. The figure sketches the decomposition from the spectral measurement data on the known reference data to retrieve concentration values at a given point in time. In the case of our measurement setup, the limiting factor for the number of gases is the number of available mass flow controllers. Without loss of generality, we restrict the number of gases in the mixture to four. This should sufficiently demonstrate the decomposition of multi-gas mixtures whilst still being economically viable. This restricts the number of basis vectors to four in this case. The setup is sealed and thus, no further vectors are necessary for this first validation. Figure 3a presents one photoacoustic spectrum as an example. The time of this measurement is marked as a green dashed line on the right. This spectrum is projected onto the reference data according to Equation (2).

The reference data $\overset{⃑}{S_{i,\mathrm{ref}},}$ is displayed in Figure 3b for each of the four different gases. Each reference spectrum is averaged ten times to reduce the influence of reference-measurement noise on retrieved concentrations in the multi-gas decomposition. This averaging does not influence the measurement time, since the undiluted-gas measurements serve only as reference data. HITRAN absorption data is plotted as a visual comparison for each gas. In some wavenumber ranges, there are minor deviations in peak height between theory data and experimental data. This can be attributed to small dips in the laser power during rapid wavelength sweeps. One could eliminate these dips by normalizing to the time-resolved laser power in a sweep. However, these dips are consistent over multiple measurements. Hence, it does not influence the accuracy of the concentration prediction.

Subsequently, spectra like the example in Figure 3a are then projected onto the basis vectors in Figure 3b. This procedure is performed for more than 20 min, acquiring one spectrum every 13 s. Linear regression retrieves the concentration coefficients *c*_i_ from the absorption data in each step. Consequently, each temporal step in Figure 3c corresponds to one recorded spectrum. The flow values in the MFCs were set such that all combinations of trace gas concentrations from 0 to 1000 ppm (C_2_H_2_, C_2_H_4_, C_2_H_6_) and 0 to 100 ppm (CH_4_), respectively, are realized in steps of five. The solid line symbolizes the concentration values that we expect from the flow ratios controlled through the mass flow controllers. It matches the measured values well. The temporal spacing proves that second-timescale monitoring is possible even for such a broad spectral range.

**Figure 4:**
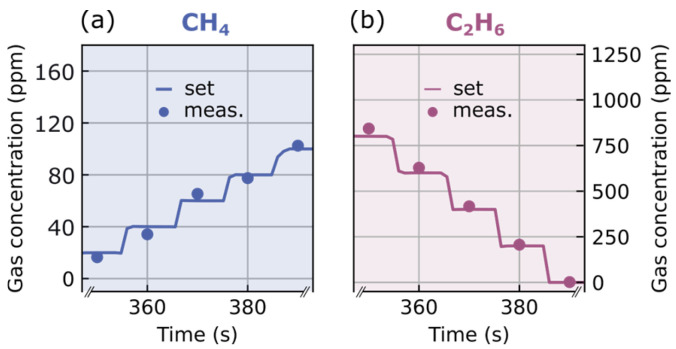
Measurement accuracy of multi-gas monitoring. In (**a**), a zoom-in on concentration values of CH_4_ shows the measurement accuracy. The same timeframe is shown in the zoom-in (**b**) for C_2_H_6_.

However, the accuracy is difficult to see solely from Figure 3. Figure 4a,b display a small subset of the measured concentrations for CH_4_ and C_2_H_2_, respectively. In this zoom-in, it becomes clear that the overlap is good between the set values and the experimentally retrieved gas contributions. The course of both values originates from the restriction that the flow into the cell is kept constant at 1 L min^−1^. As the concentrations of C_2_H_2_ and C_2_H_4_ are kept constant in the selected timeframe (350–390 s), the concentration of C_2_H_6_ needs to be lowered to increase the concentration of CH_4_ while maintaining constant flow.

The number of gases that are simultaneously distinguishable is governed by spectral bandwidth and resolution. A larger spectral range allows coverage of more spectral features, and a narrower linewidth of the laser allows finer selection of individual absorption features. This raises the question of how many gases could be distinguished theoretically. We have tested this on one mixture spectrum by narrowing the bandwidth for concentration retrieval. The retrieval is robust for the mixture of four gases until the bandwidth is restricted to below 8 cm^−1^. Then, the relative error starts to increase. Details can be found in Figure A7. Thus, we conclude that for our sampling of 1 cm^−1^, the number of gases retrievable is half of the wavenumber span. As our wavenumber span is over 400 cm^−1^, more than 200 gases could be distinguished. This is further restricted by the availability of absorption features in a given wavelength range.

**Figure A1:**
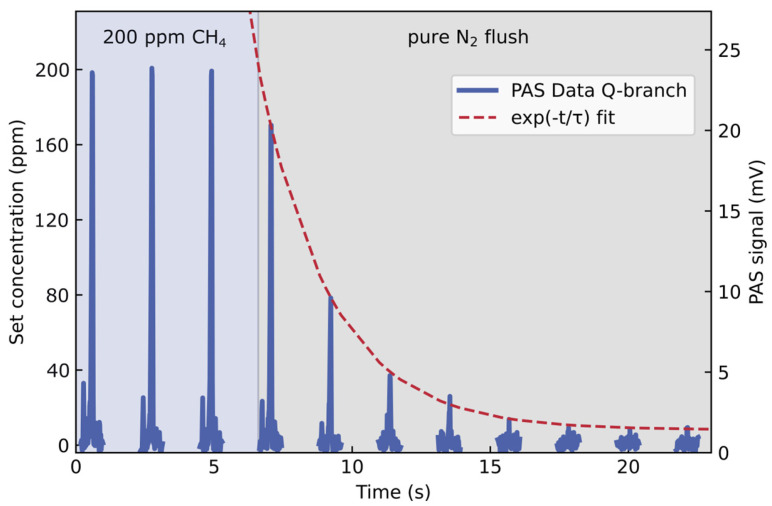
Speed limitations of dynamic gas monitoring using QEPAS. For dynamic sensing applications, it is important to estimate how quickly changes in concentration can be monitored. For this, the flow is rapidly changed from 200 ppm CH_4_ to pure N_2_ (black dots and line) at a constant flow rate of 2 L min^−1^. The cell itself holds a volume of 7 cm^3^. From a naïve mathematical standpoint, not considering any sort of gas dynamics, the cell should therefore be flushed in less than two seconds. We perform consecutive sweeps on the Q-branch of CH_4_ from 3300 cm^−1^ to 3345 cm^−1^ (violet). The sensing of residual gas signal without distortion due to fork ringing is only possible due to active deceleration of the fork. The height of the main branch, indicated by blue crosses, decreases much slower than the setpoints of the mass flow controllers. The decrease follows an exponential decay e^−t/τ^ with a time constant of τ = 4.3 s as indicated by the red dashed line. We attribute this behavior to gas dynamics in the sensing volume, as remainders of the initial gas are present even after continuous flushing. Therefore, the cell volume restricts measurement time, not the spectral analysis itself.

The relatively high flow of 1 L min^−1^ was chosen to gauge the ability to measure dynamic gas changes. The equilibrium dynamics inside the cell become a limiting factor in measurement speed at the data acquisition rate of a few seconds. Although the flow exceeds the recommended maximum flow value of 0.2 L min^−1^ stated by the manufacturer, the experimental data is not affected greatly by the flow-induced noise. The noise level for higher flow is around 300 µV, which is an increase by a factor of 3 from the noise level when the flow is within the specified range. When the expected concentrations are large and the dynamic changes are of interest instead of the absolute limit, this is a compromise worth making. It is noteworthy that the measurement cell volume of 7 cm^3^ is already small compared to other gas detection methods. To quantify the limitation of gas composition equilibrium in the cell with respect to the incoming flow, we change the flow from 200 ppm CH_4_ to pure N_2_ at 2 L min^−1^ within a few milliseconds. Meanwhile, we perform sweeps on the strongest peak of methane, the Q-branch. In this way, we can monitor changes in gas concentration in the cell not superimposed by fork ringing. At each individual wavenumber measurement point, the fork is stopped completely. Measurements are taken only on the photoacoustic transients. The signal follows an exponential decay with τ = 4.3 s and a characteristic T_90_ time of 9.9 s. Details are provided in Figure A1. This decay is in the same order of magnitude as the sweeping time, i.e., the time it takes to acquire one spectrum of a gas mixture. Hence, sweeping even faster would not enable higher accuracy. Moreover, in the case of monitoring gas components, the gas dynamics outside of the cell are likely to be on the same timescale. With the temporal measurement accuracy (“*x*-axis”) now quantified, we turn our attention to the accuracy of concentration estimation (“*y*-axis”).

**Figure 5:**
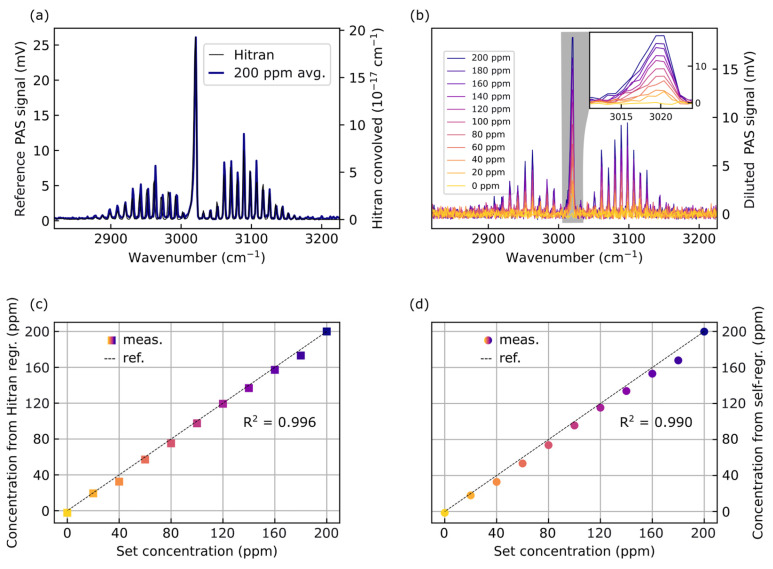
Linear regression for concentration analysis. For the sake of simplicity and without loss of generality, we show a single gas species (CH_4_). Panel (**a**) features the (averaged) basis vector of 200 ppm CH_4_ in a N_2_ matrix (blue) compared to HITRAN data (black). Spectra of these diluted trace gases are shown in (**b**). The decrease in signal amplitude is clearly visible, especially in the Q-branch inset. Linear regression is depicted in (**c**) for a mapping to HITRAN data and in (**d**) for self-regression by simple mapping on the basis vector shown in (**a**). Both regression methods fit yield only minute differences to the concentrations set by the mass flow controllers indicated by the dashed line.

To do so, we use a single gas species—methane—and dilute it. Then, the retrieved concentrations serve to estimate how accurately we can determine a given concentration of a gas. Figure 5 displays the linear regression results for methane dilution from 200 ppm to 0 ppm in steps of 20 ppm. In Figure 5a, the reference vector taken at 200 ppm is plotted against expected HITRAN data convolved with the laser linewidth of 2 cm^−1^. Both agree well visually. We also note a noise background with a base level of 300 µV. Figure 5b shows the spectra corresponding to each dilution level. Again, the reference vector is averaged over ten measurements whereas the individual sweeps are not. This means that sampling a single concentration value only takes about four seconds for the swept wavenumber range.

In the bottom row of Figure 5, two different regression strategies are evaluated. In Figure 5d, we continue to use the self-contained approach: The spectrum at 200 ppm is used directly as the reference spectrum. The coefficient of determination is R^2^ = 0.990. One could also construct a slightly different version of linear regression that incorporates HITRAN data directly, depicted by Figure 5c. There, the power spectral densities (PSDs) of the measured 200 ppm-signal and the HITRAN spectrum are integrated and related via a conversion factor. Then, the HITRAN data is used as a reference vector instead of the experimentally measured reference spectrum. The coefficient of determination with this method is R^2^ = 0.996.

**Figure A2:**
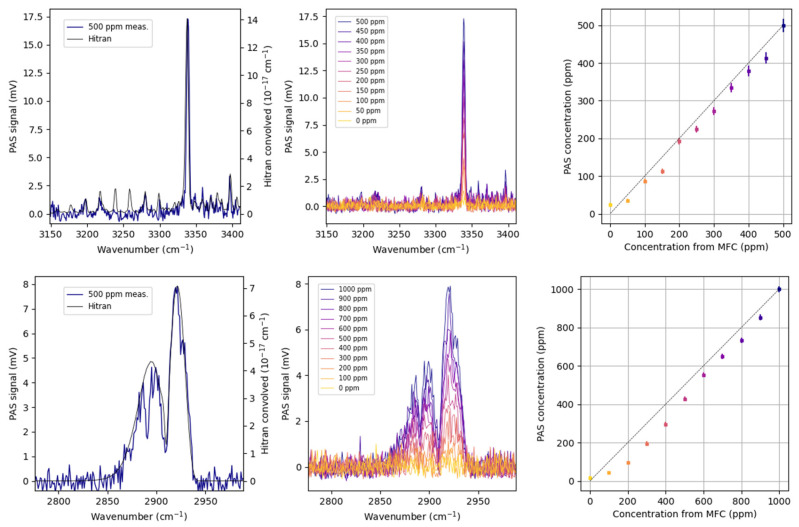
Linear regression results on NH_3_ and NO_2_. The top row shows NH_3_, the bottom row NO_2_. In both rows, the left plot compares the basis vector to theoretical HITRAN data. The middle plot shows the spectra of diluted trace gases. From this data, the concentrations are extracted via linear regression on the right. We note that the extracted concentrations are slightly underestimated.

Visually, both methods yield consistent results across the entire dilution range. Each method has its advantages. A direct comparison using only measured data is elegant as it does not require prior knowledge from an external database and is therefore self-contained. Yet, converting the signal using the PSD would allow estimation and identification of previously unknown constituents via the HITRAN database in the future. Using the method in Figure 5c, unknown gas constituents could be mapped onto existing convolved spectra by linear regression using the HITRAN-derived reference vector. In the measurements reported here, all the gases are known. Accordingly, we restrict the evaluation to the self-contained method. Additional data for validation of the linearity in the regression is performed on NH_3_ and NO_2_. These results are plotted in Figure A2. Furthermore, we validate the linear dependence of the spectral response by varying the laser power in Figure A8 with R^2^ = 0.997.

After validating the ability of COCO-QEPAS to monitor gas mixtures, it is interesting to test the detection limit of spectral fingerprinting. We do this to display the ability of COCO-QEPAS to sense not only quickly, but also with good accuracy at the cost of measurement speed. To do so, we dilute methane even further down to 10 ppm.

It is nontrivial to quantify the detection limit then. One can start off with a simple peak-to-valley comparison. Here, we compare the highest absorption in the Q-branch with the region around 2840–2860 cm^−1^ where the convolved HITRAN absorption data lies below 0.05 cm^−1^, which is less than 0.3% of the maximum value at the Q-branch.

**Figure 6:**
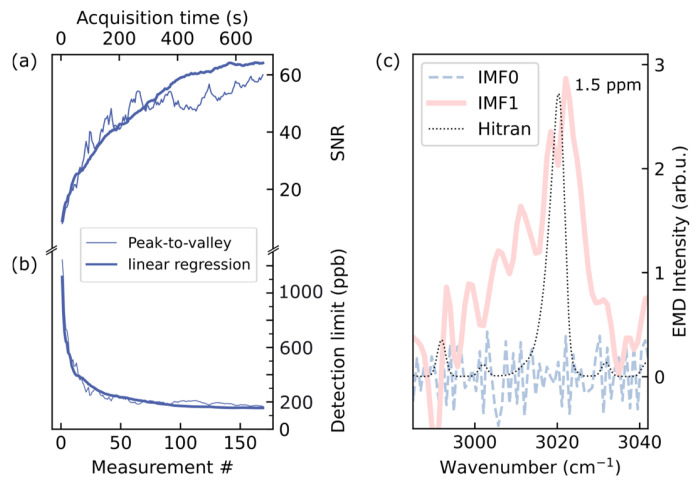
Quantification of detection limits. The left panels depict the signal improvement for averaging multiple recorded spectra. Subfigure (**a**) shows the signal-to-noise ratio (SNR) as a function of acquisition time. In fact, the x-axis for both (**a**) and (**b**), where we plot the detection limit, are shared as each spectrum takes 4.2 s acquisition time. Hence, the number (#) of averaged spectra can be deducted from the lower x-axis. In (**c**), we show that it is possible to retrieve a meaningful spectral fingerprint in the ppb-range using empirical mode decomposition from its intrinsic mode 1 function (IMF1).

Instead of simple peak-to-valley comparison, it is also possible to use the data of all branches (P, Q and R) using linear regression. The error is plotted as thick line in Figure 6b. The higher amount of data points allows for a smooth course of the curve over the number of averaged spectra. This is also the case for the detection limit in Figure 6c. The SNR, calculated by dividing the retrieved concentration value by the standard deviation of the average value of an absorption-free spectral region, shows a √n behavior for the linear regression on a 200 ppm reference and also, for the simple peak-to-valley ansatz. The highest SNR is around 60 for both methods. The valley is taken from the 2840 to 2860 cm^−1^ region with a noise floor of 30 µV, where no methane absorption is present, and the peak is taken as maximum signal of the Q-branch around 3017 cm^−1^. Considering that the detection limit is reached when the peak vanishes in the noise, we can calculate it by dividing the measured concentration by the SNR, i.e., the concentration at which one would obtain SNR = 1. The detection limit also decreases as a function of 1/√n for both methods with a minimal detection limit of 90 ppb. We can see that the detection limit is already reduced by a factor of 3 (from 1200 ppb to below 400 ppb) after 45 s of acquisition time. For sensing in low-concentration regimes, this could be a compromise between measurement speed and accuracy. The absorption at this fingerprint is plotted in Figure A9 for an average of 170 measurements.

**Figure A4:**
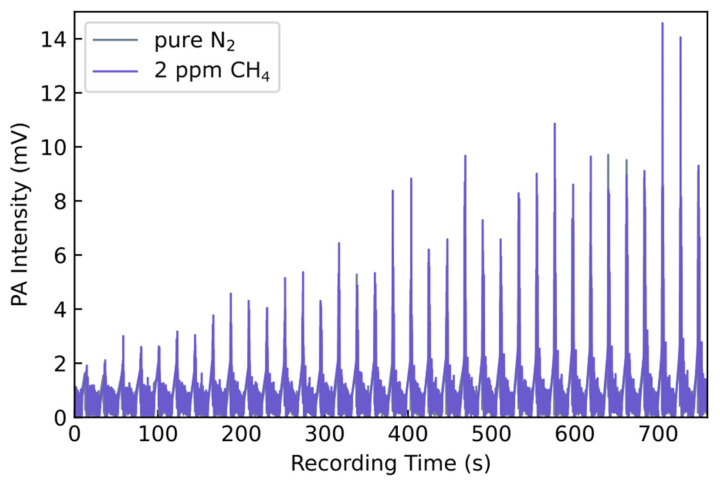
Background increase for low-concentration measurements. Shown are two measurement series of spectra over time, with 2 ppm CH_4_ and with pure N_2_ in the cell. Over time, water and carbon dioxide start to leak into the cell and vaporize from the cell walls. This leads to an increase in signal at the water wavelengths. We can subtract these contributions to retrieve signals at very low concentrations.

For concentrations below 2 ppm, conventional regression methods become increasingly limited by noise. In this regime, empirical mode decomposition (EMD) can be applied to averaged signals to recover characteristic spectral features [52]. Figure 6c illustrates this for methane at 1.5 ppm, where the Q-branch is extracted from the first intrinsic mode function (IMF1). Despite significant peak broadening caused by the low signal-to-noise ratio, the spectral position and shape remain consistent with the HITRAN reference, confirming the validity of the retrieval. This dataset was obtained from 33 averaged spectra, corresponding to an acquisition time of approximately two minutes (see Figure A4 for details). While EMD is traditionally applied to time-domain signals, it can also be effectively used for spectroscopic data treated as one-dimensional signal as a function of wavenumber. The key idea is that EMD decomposes a signal into intrinsic mode functions (IMFs) according to local frequency content and amplitude modulations. In the case of noisy absorption spectra, high-frequency IMFs predominantly contain random noise, while lower-order IMFs retain the smoother, physically meaningful spectral structures. Thus, EMD acts as a data-driven, adaptive filter that can separate the methane Q-branch signature from broadband noise without assuming a predefined basis, unlike Fourier or wavelet filtering. This enables recovery of weak spectral features even when the SNR is too low for reliable conventional regression.

Since the relevant signal components overlap with baseline drift in both time and frequency, linear time-invariant filtering approaches such as Savitzky–Golay or wavelet filtering cannot achieve meaningful component separation without prior assumptions on signal structure. Therefore, adaptive, data-driven decomposition is required, which motivates the use of EMD in the present work.

EMD thus provides a complementary post-processing technique that enables trace detection in the single-digit ppm regime, where linear regression alone no longer yields reliable results. While less suited for absolute quantification, it is a powerful tool for qualitative spectral identification and for extending the detection capability of COCO-QEPAS towards the ppb range.

**Figure 7:**
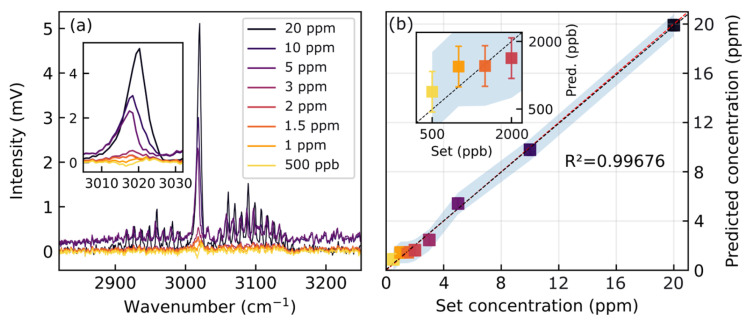
Principal component regression for concentration retrieval in CH_4_ below 20 ppm. (**a**) Plots the retrieval for two loadings at concentrations from 20 ppm to 250 ppb. The retrieved concentration values are displayed in (**b**), with error bars indicating 1-sigma deviation and the shaded area a 95% confidence interval. Even down to the ppb level, concentrations can be retrieved with good accuracy (R^2^ = 0.99686).

**Figure A6:**
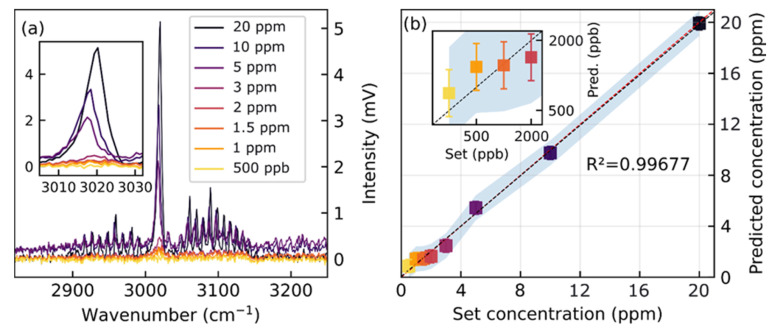
Principal component regression for concentration retrieval in CH_4_ below 20 ppm with 3 loadings. (**a**) Plots the retrieval for two loadings at concentrations from 10 ppm to 250 ppb. The retrieved concentration values are displayed in (**b**), with error bars indicating 1-sigma deviation and the shaded area a 95% confidence interval.

**Figure A7:**
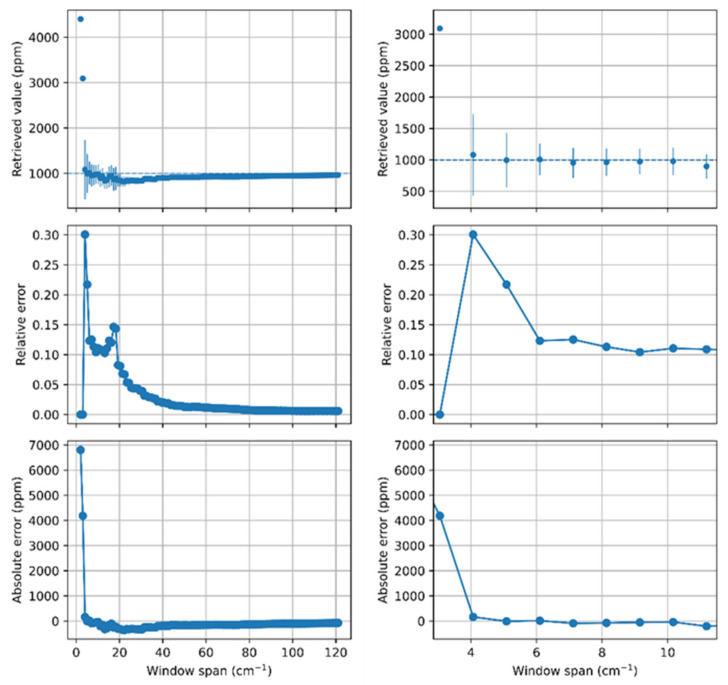
Test of regression stability on a four-gas mixture spectrum. The regression stability only decreases after narrowing the spectrum below 10 cm^−1^.

**Figure A8:**
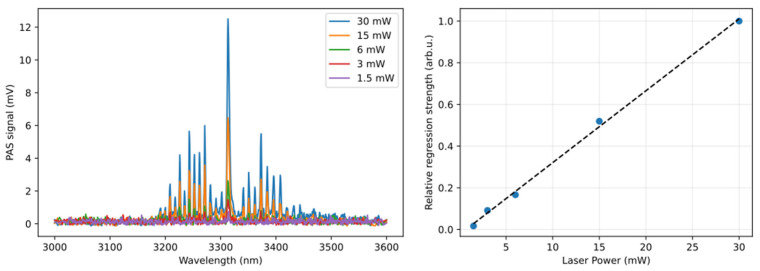
Linear regression with varying laser power. When the laser power decreases, the magnitude of the spectra decreases linearly. We note an R^2^ value of 0.997 for the regression based on the spectrum at 30 mW.

**Figure A9:**
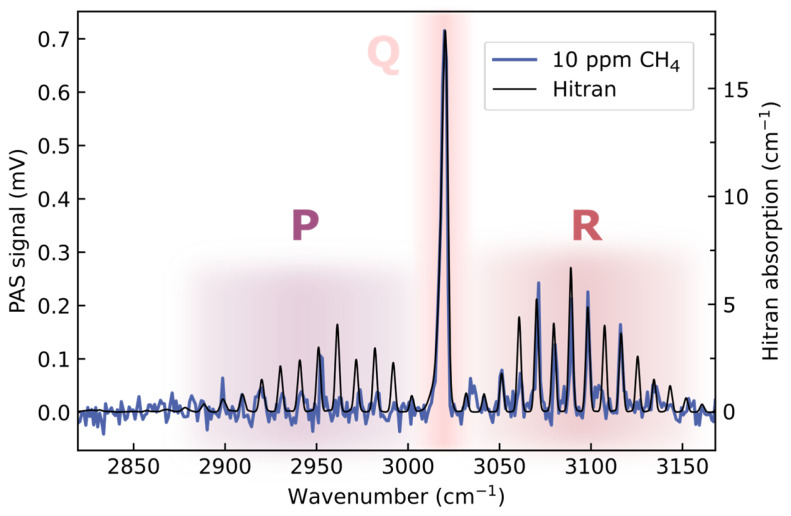
Averaged spectral fingerprint at 10 ppm. The blue line shows the 170-times averaged signal, with the respective absorption branches (P, Q and R) indicated visually.

At lower concentrations, the signal-to-noise ratio becomes increasingly insufficient for reliable quantification using univariate linear regression. Accordingly, the analysis in Figure 5 was restricted to concentrations between 200 ppm and 20 ppm. To overcome this limitation, we apply principal component analysis (PCA), which exploits the full spectral information by reducing it to a small number of dominant loadings. These loadings serve as input variables for a multivariate regression model, which effectively represents the inverse sensing or calibration problem—that is, reconstructing the gas concentration from the measured spectrum. In contrast to a direct fit of individual spectral features, principal component regression [53] captures correlated variations across the entire spectrum, thereby enhancing sensitivity in the low-concentration regime while suppressing noise contributions that would otherwise dominate single channels. Figure 7 illustrates the performance of this approach below 20 ppm. Already at two principal components, the retrieved concentrations show good agreement with the measurements (R^2^ = 0.9969), which only slightly deviates towards the ppb range. For validation, we note that the results obtained with three components are nearly indistinguishable from those with two, confirming that higher-order loadings are not required, plotted in Figure A6. The choice of two principal components is supported both empirically and theoretically: the first two components account for more than 99% of the total variance, representing the dominant absorption features and their linear scaling with concentration, while higher-order components mainly reflect uncorrelated noise and baseline drift. Retaining only these two components ensures that the inverse model captures the physically relevant variance associated with methane absorption while suppressing noise-driven artifacts. We assume that the over-estimation of the retrieved concentrations at 500 ppb and below stems from intramolecular relaxation becoming more apparent due to water leakage over the averaging process, which otherwise is not of relevance according to Figure 12 in [54].

In general, significant humidity has an influence on the relaxation rates when sampling ambient air. On the one hand, relaxation rates become relevant. On the other hand, water vapor can be responsible for dips in the laser power over a given spectral range before the laser beam even reaches the detection cell. For future field deployment of the system, the travel distance of the laser beam should therefore be kept to a minimum and humidity needs to be monitored. The monitoring could also be performed on a distinct absorption line of water itself.

**Figure 8:**
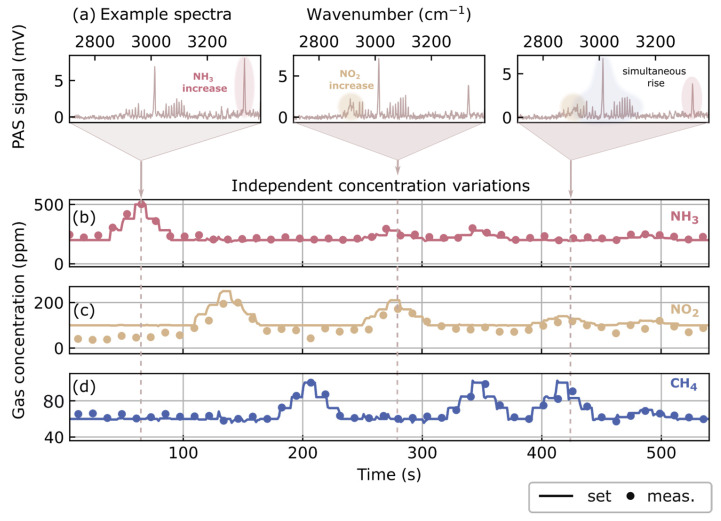
Independence of concentration variations in gas mixtures. A different set of gases is individually and simultaneously modulated. Panel (**a**) depicts three exemplary absorption spectra. Characteristic elevated fingerprints of each gas are highlighted. Panels (**b**–**d**) present the derived concentrations for each gas. The concentrations are individually modulated (“bumped”) first, then modulated sequentially in various combinations, indicating independent monitoring.

Although this demonstrates the validity of the concept for a single gas at different concentrations, one might still raise the question of whether the extension to multiple gases in a linear fashion is valid. Hence, in Figure 8 the independence of concentration variations is tested. The gases considered are CH_4_, NH_3_, and NO_2_. In addition, this expands the set of measured gas mixtures. The three panels in Figure 8a display exemplary spectra of the mixture. In these spectra, elevated signals at the characteristic fingerprints for each gas are highlighted. The data in Figure 8b–d corresponds to the retrieved concentration coefficients for CH_4_, NO_2_, and NH_3_, respectively.

Concentration variations in the three gases are probed in different combinations. First, the concentration is raised and lowered individually for each gas, whereas the other two are kept constant. Subsequently, the concentrations are raised and lowered simultaneously for two gases. Eventually, all three gases are varied concurrently. The fourth MFC is connected to a pure nitrogen line which allows the other concentrations to be varied freely, in contrast to the four-gas mixture in Figure 3 and Figure 4.

NH_3_ measurements are nontrivial due to its tendency to adsorb and react with surfaces in the measurement environment, which explains the initial deviation in NO_2_ values when NH_3_ is varied most strongly. Nonetheless, the concentrations in mixtures can be retrieved individually without cross-interference. This also demonstrates that intramolecular relaxation processes do not influence the measurement results significantly despite the spectral overlap of the absorption bands.

## 4. Conclusions

This paper presents coherently controlled QEPAS (COCO-QEPAS) measurements for analyzing and monitoring arbitrary trace gas mixtures. We have demonstrated detection limits (SNR = 1) for full spectral fingerprints below a concentration of 180 ppb CH_4_ in N_2_ for a sweep time of 640 s (170 sweeps at 4 s each) at a laser power of 100 mW with a spectral laser linewidth of 2 cm^−1^. From there, the sensing capability remains linear up to percent-level concentrations for gas mixtures—with second-scale precision, shown for up to four gases simultaneously, and straightforward scalability. This scalability should enable time-resolved trace gas measurements in ambient atmosphere in the future, allowing multi-component monitoring. The dynamic response, small sampling volume, high sensitivity, and high specificity make our method ideal for sensing volatile organic compounds (VOCs) and catalyst gases. Applications include breath analysis, industrial process control, and petrochemical analysis. Moreover, since the absorption in the mid-infrared region is responsible for the greenhouse effect, sensing in this spectral range is ideal for environmental monitoring.

## Figures and Tables

**Table 1:** Comparison of recent QEPAS results in relevant performance parameters.

Ref.	Sampling Time	Detection Limit	Number of Gases
[46]	3.1 s	1 ppm	1
[47]	3 s	875 ppb	2
[48]	8 ms–1000 s	10 ppm	1
[49]	3 ms	40 ppm	1
This work	3–45 s	450 ppb	4 (demonstrated)

**Figure A3:**
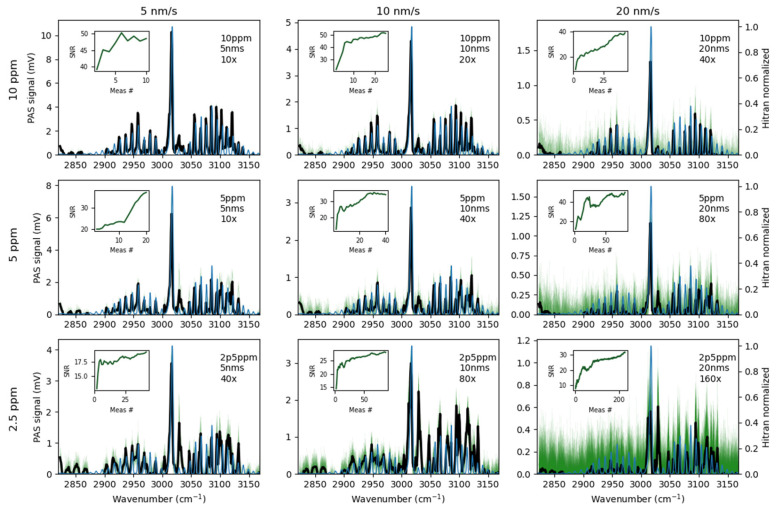
Exploring the parameter space and its limitations for single-digit ppm fingerprints. Trace gas concentration, sweeping speed and number of integrations are varied on a CH_4_ measurement such that they should result in a similar SNR. The SNR does decrease slightly stronger with lower concentrations. This can be explained by residual trace gases and water vapor that are still present in the cell. Also, faster sweeping and more averaging is the optimal distribution of available measurement speed.

**Figure A5:**
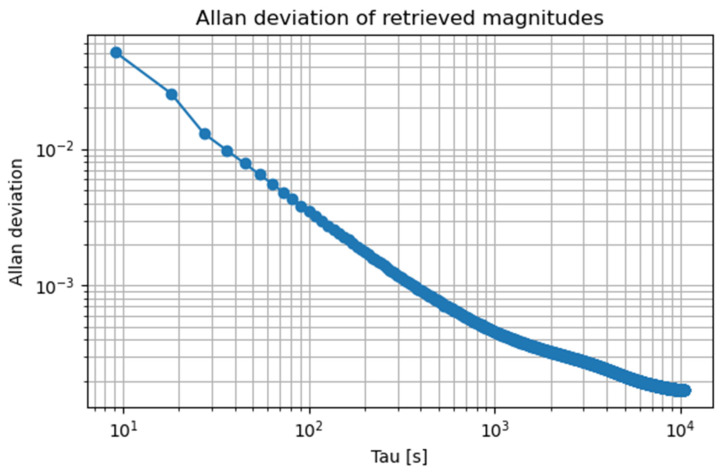
Allan deviation at 200 ppm concentration. For several mappings of the averaging onto the reference vector, the deviation decreases. This decrease is getting less over time and starts to converge for an averaging time of over 104 s.

## Data Availability

The raw data supporting the conclusions of this article will be made available by the authors on request.

## Abbreviations

The following abbreviations are used in this manuscript:

AOM	Acousto-optic modulator
COCO-QEPAS	Coherent control in quartz-enhanced photoacoustics
CH_4_	Methane
C_2_H_2_	Acetylene
C_2_H_4_	Ethylene
C_2_H_6_	Ethane
PAS	Photoacoustic spectroscopy
SNR	Signal-to-noise ratio
ppm	Parts per million
ppb	Parts per billion
CRDS	Cavity-ringdown spectroscopy
VOCs	Volatile organic compounds
ECDL	External cavity diode lasers
EMD	Empirical mode decomposition
PCA	Principal component analysis
DCS	Dual-comb spectroscopy
FTIR	Fourier-transform infrared spectroscopy
QCL	Quantum cascade laser
PSD	Power spectral density
IMF	Intrinsic mode function
AWG	High-transmission
DAQ	Data acquisition
MFC	Mass flow controller
FFOPO	Fiber-feedback optical parametric oscillator
HITRAN	High-resolution transmission molecular absorption database
